# DNorm: disease name normalization with pairwise learning to rank

**DOI:** 10.1093/bioinformatics/btt474

**Published:** 2013-08-21

**Authors:** Robert Leaman, Rezarta Islamaj Doğan, Zhiyong Lu

**Affiliations:** ^1^National Center for Biotechnology Information, 8600 Rockville Pike, Bethesda, MD 20894, USA and ^2^Department of Biomedical Informatics, Arizona State University, 13212 East Shea Blvd, Scottsdale, AZ 85259, USA

## Abstract

**Motivation:** Despite the central role of diseases in biomedical research, there have been much fewer attempts to automatically determine which diseases are mentioned in a text—the task of disease name normalization (DNorm)—compared with other normalization tasks in biomedical text mining research.

**Methods:** In this article we introduce the first machine learning approach for DNorm, using the NCBI disease corpus and the MEDIC vocabulary, which combines MeSH® and OMIM. Our method is a high-performing and mathematically principled framework for learning similarities between mentions and concept names directly from training data. The technique is based on pairwise learning to rank, which has not previously been applied to the normalization task but has proven successful in large optimization problems for information retrieval.

**Results:** We compare our method with several techniques based on lexical normalization and matching, MetaMap and Lucene. Our algorithm achieves 0.782 micro-averaged F-measure and 0.809 macro-averaged F-measure, an increase over the highest performing baseline method of 0.121 and 0.098, respectively.

**Availability:** The source code for DNorm is available at http://www.ncbi.nlm.nih.gov/CBBresearch/Lu/Demo/DNorm, along with a web-based demonstration and links to the NCBI disease corpus. Results on PubMed abstracts are available in PubTator: http://www.ncbi.nlm.nih.gov/CBBresearch/Lu/Demo/PubTator

**Contact:** zhiyong.lu@nih.gov

## 1 INTRODUCTION

Diseases are central to many lines of biomedical research, and enabling access to disease information is the goal of many information extraction and text mining efforts (Islamaj Doğan and Lu, 2012b; Kang *et al.*, 2012; Névéol *et al.*, 2012; Wiegers *et al.*, 2012). The task of disease normalization consists of finding disease mentions and assigning a unique identifier to each. This task is important in many lines of inquiry involving disease, including etiology (e.g. gene–disease relationships) and clinical aspects (e.g. diagnosis, prevention and treatment).

Disease may be defined broadly as ‘any impairment of normal biological function’ (Hunter, 2009). Given the wide range of concepts that may thus be categorized as diseases—their respective etiologies, clinical presentations and their various histories of diagnosis and treatment—disease names naturally exhibit considerable variation. This variation presents not only in synonymous terms for the same disease, but also in the diverse logic used to create the disease names themselves.

Disease names are often created by combining roots and affixes from Greek or Latin (e.g. ‘hemochromatosis’). A particularly flexible way to create disease names is to combine a disease category with a short descriptive modifier, which may take many forms, including anatomical locations (‘breast cancer’), symptoms (‘cat-eye syndrome’), treatment (‘Dopa-responsive dystonia’), causative agent (‘staph infection’), biomolecular etiology (‘G6PD deficiency’), heredity (‘X-linked agammaglobulinemia’) or eponyms (‘Schwartz-Jampel syndrome’). Modifiers are also frequently used to provide description not part of the name (e.g. ‘severe malaria’).

When diseases are mentioned in text, they are frequently also abbreviated, exhibit morphological or orthographical variations, use different word orderings or use synonyms. These variations may involve more than single word substitutions. For example, because affixes are often composed, a single word (‘oculocerebrorenal’) may correspond to multiple words (‘eye, brain and kidney’) in another form.

The disease normalization task is further complicated by the overlap between disease concepts, forcing systems that locate and normalize diseases in natural language text to balance handling name variations with differentiating between concepts to achieve good performance. Previous works addressing disease name normalization (DNorm) typically use a hybrid of lexical and linguistic approaches (Islamaj Doğan and Lu, 2012b; Jimeno *et al.*, 2008; Kang *et al.*, 2012). While string normalization techniques (e.g. case folding, stemming) do allow some generalization, the name variations in the lexicon always impose some limitation. Machine learning may enable higher performance by modeling the language that authors use to describe diseases in text; however, there have been relatively few attempts to use machine learning in normalization, and none for disease names.

In this work we use the NCBI disease corpus (Islamaj Doğan and Lu, 2012a), which has recently been updated to include concept annotations (Islamaj Dogan *et al.*, unpublished data), to consider the task of disease normalization. We describe the task as follows: given an abstract, return the set of disease concepts mentioned. Our current purpose is to support entity-specific semantic search of the biomedical literature (Lu, 2011) and computer-assisted biocuration, especially document triage (Kim *et al.*, 2012).

In this article we introduce DNorm, the first machine learning method to normalize disease names in biomedical text. Our technique learns the similarity between mentions and concept names directly from the training data, thereby focusing on the candidate generation phase of normalization. Our technique can learn arbitrary mappings between mentions and names, including synonymy, polysemy and relationships that are not 1-to-1. Moreover, our method specifically handles abbreviations and word order variations. Our method is based on pairwise learning to rank (pLTR), which has been successfully applied to large optimization problems in information retrieval (Bai *et al.*, 2010), but to the best of our knowledge has not previously been used for concept normalization.

### 1.1 Related work

Biomedical named entity recognition (NER) research has received increased attention recently, partly owing to BioCreative (Hirschman *et al.*, 2005b) and BioNLP (Kim *et al.*, 2009) challenges on recognition of genes, proteins and biological events in the scientific literature, as well as TREC (Voorhees and Tong, 2011) and i2b2 (Uzuner *et al.*, 2011) challenges on identification of drugs, diseases and medical tests in electronic patient records.

The problem of concept normalization has seen substantial work for genes and proteins, as a result of a series of tasks that were part of the BioCreative competitions (Hirschman *et al.*, 2005a; Lu *et al.*, 2011; Morgan *et al.*, 2008). A variety of methods including pattern matching, dictionary lookup, machine learning and heuristic rules were described for the systems participating in these challenges. Articles have also discussed the problem of abbreviation definition and expansion, rule-based procedures to resolve conjunctions of gene names, lexical rules to address term variation in gene names, enhanced dictionaries, approximate string matching and filtering approaches to reduce false positives.

A large portion of concept normalization work relies, at least partially, on dictionary lookup techniques and various string matching algorithms to account for term variation. Although machine learning components have been implemented, the majority of the investment in this line of work has been the establishment of various filtering techniques to select the right candidates for normalization. For example, Buyko *et al.* (2007) used conditional random fields to solve the problem of gene mention coordination, Tsuruoka *et al.* (2007) used a logistic regression method for learning a string similarity measure from a dictionary and Wermter *et al.* (2009) incorporate a semantic similarity scoring module in their GeNo gene-name normalization system. Listwise learning to rank techniques, which learn the best list of objects to return rather than the best single object, have been used for gene name normalization in Huang *et al.* (2011a) and in MeSH® term selection for indexing in Huang *et al.* (2011b). While the listwise approach is useful when the notion of relevance for the task is multifaceted or involves varying degrees of relevance, in this work we use pLTR because our interest is in the single best name for each mention. Recently, Islamaj Doğan and Lu (2012b) successfully built a rule-based inference method with application to disease name normalization to MeSH and OMIM terminology.

Disease name recognition and disease concept identification has received less attention when compared with other biomedical concept recognition tasks, possibly owing to the fact that there is no gold standard that can be used to evaluate existing techniques and/or build new ones focusing on the identification of diseases in text. Several terminology resources are available that provide disease terms, such as MeSH, National Cancer Institute thesaurus, SNOMED-CT (Stearns *et al.*, 2001), UMLS, Disease Ontology (Schriml *et al.*, 2012) and MEDIC (Davis *et al.*, 2012). The UMLS Metathesaurus covers much more than any of the other resources because its main purpose is the comprehensive coverage of medical terminology terms. The UMLS was used in the corpus developed by Jimeno *et al.* (2008), which evaluated several normalization methods at the sentence level; the highest performing method was a dictionary lookup method, which achieved 0.684 in F-measure. The corpus of Jimeno *et al.* was then extended by Leaman *et al.* (2009), and subsequently used by Kang *et al.* (2012) to achieve an F-measure of 0.736 on concept identifier matching.

Recently, a new disease lexicon, namely MEDIC (Davis *et al.*, 2012), was created by the Comparative Toxicology Database for indexing diseases in biomedical literature during biocuration. MEDIC merges OMIM into the disease branch of MeSH, making it a natural choice for indexing purposes, and is therefore used as the lexicon for the NCBI disease corpus (Islamaj Doğan and Lu, 2012a), which consists of nearly 800 PubMed abstracts manually annotated with respect to diseases. Such a corpus provides a large-scale resource for enabling the development of more precise tools that address disease name recognition and normalization.

## 2 METHODS

We use the NCBI disease corpus, which consists of 793 PubMed abstracts, split into three subsets as described in Table 1. Each abstract was annotated by two human annotators for disease mentions, as well as their corresponding concept identifiers in MEDIC (inter-annotator agreement: 87.5%). Each abstract contains an average of 5.08 disease mentions and 3.28 disease concepts. In this research, we use the December 6, 2012 version of MEDIC, which contains 11 583 MeSH identifiers and 3990 OMIM identifiers, grouped into 13 339 disease concepts. This version contains 75 761 names, including synonyms. The average number of names per concept is 5.72 and the average number of concepts per name is 1.01.

**Table 1.** Size of the NCBI disease corpus

Setup	Abstracts	Mentions	Concepts
Training subset	593	5145	670
Development subset	100	787	176
Test subset	100	960	203

### 2.1 Processing pipeline

**Fig. 1.**
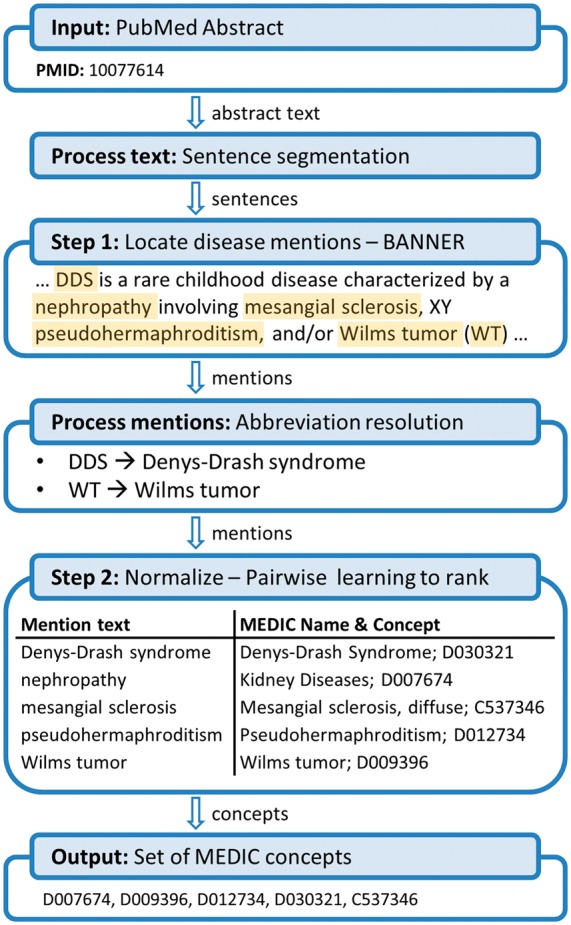
The DNorm disease normalization pipeline, with examples, as described in Section 2.1

We process PubMed abstracts using a pipeline architecture summarized in Figure 1. Abstracts are processed first by breaking into sentences using the built-in Java class for sentence segmentation. We improved the accuracy of the segmentation by disallowing sentence breaks within parenthesis.

Disease mentions are then located using the BANNER named entity recognizer (Leaman and Gonzalez, 2008). BANNER is a trainable system, using conditional random fields (Lafferty *et al.*, 2001) and a rich feature set approach. As in previous work for disease name recognition, our feature set that included a dictionary of disease names derived from the UMLS Metathesaurus (Leaman *et al.*, 2009). For this project, we used a BANNER model trained on the training subset of the NCBI disease corpus.

Mentions output by BANNER are then subjected to additional string processing. Abbreviation definitions are located in the abstract (Sohn *et al.*, 2008), and short-form abbreviations found in mentions are replaced with their long form. If the mention already includes the long form, however, the abbreviation is instead dropped. Mentions are then tokenized at whitespace and punctuation. Punctuation and stop words listed in the default set of English stop words in the information retrieval library Lucene (http://lucene.apache.org) are removed, while digits are retained. Tokens are then converted to lower case ASCII and stemmed using the Porter stemmer implementation provided by Lucene.

The next step is to generate candidate concepts for each mention. Our method finds the best match between the mention and the disease names in MEDIC by defining a vector space, converting both mentions and concept names to vectors within that space, then searching for the name that maximizes a scoring function learned from the training data. We describe our technique in detail in Section 2.2 and Section 2.3.

The final step before returning results is disambiguation: we identify whether the name is listed as the primary name or a synonym for the disease concept, and filter matches to a synonym if a parent uses the same name as a primary name. After filtering, we return the disease concept associated with the highest scoring name, breaking ties arbitrarily.

### 2.2 Pairwise learning to rank

We formalize the normalization problem as follows: Let 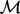 represent a set of mentions from the corpus, 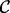 represent a set of concepts from a controlled vocabulary such as MEDIC and 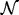 represent the set of concept names from the controlled vocabulary (the lexicon). We assume that each mention 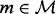 in the dataset is annotated with exactly one concept 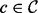. We also assume that the controlled vocabulary describes a many-to-many mapping between concepts 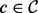 and names 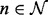.

To represent these relationships, we define the function 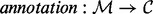 such that given a mention 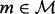, it returns the annotated concept 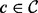 in the dataset. We also define 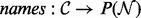, where 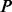 is the power set function, so that given a concept 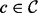, it returns the subset of 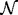 specified in the controlled vocabulary as the set of names associated with 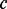.

Under these definitions, the candidate generation task can be modeled as the task of ranking pairs of mentions and concept names. We create a function that returns a numeric score for any tuple 〈
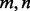
〉, 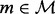, 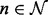, that is 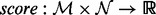. We can then generate candidate concepts for a given mention by iterating through all names, finding the name with the highest score and returning the associated disease concept.

The primary effort therefore becomes the creation of an appropriate scoring function. We use the training data to learn a function that will return a higher score for matching pairs than for mismatched pairs. That is, given mention 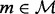, concept 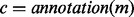, name 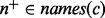 and name 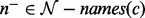, we would like a scoring function that generally obeys the constraint 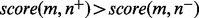 (Bai *et al.*, 2010).

We define a set of tokens 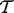 containing the tokens from all mentions 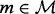 and all names 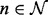. We define a vector space of dimensionality 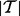 and represent both mentions and names as Term Frequency-Inverse Document Frequency (TF-IDF) vectors within that space (Manning *et al.*, 2008). We calculate the TF for each element in the vector as the number of times the corresponding token appears in the mention or name. The IDF for each element in mention and name vectors is calculated from the number of names in the lexicon that contain the corresponding token, as follows:

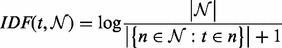



All vectors are normalized to unit length. To simplify the notation, we use 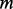 to represent both the token list form and the TF-IDF vectors of mentions, and 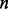 to represent the same for names.

In this work we choose the scoring function to be a linear function of all possible pairs of tokens between mention 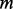 and name 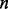. We introduce a matrix, 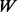, to contain the weights of the linear function, and express the scoring function in matrix form as:

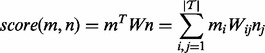



In this model, entry 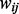 in the weight matrix 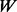 represents the correlation between token 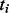 appearing in a mention and token 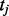 appearing in a concept name from the lexicon. This model has several useful properties. It is capable of representing both positive and negative correlations between tokens, and models both synonymy and polysemy. The model also does not assume that the token distributions are the same between the mentions and the names.

Our method finds the best potential matches for mention 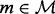 by iterating through all 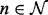 and then passing the names with the highest values for 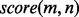 to the disambiguation component. We also set a threshold so that names given a score less than or equal to 0 are not returned.

### 2.3 Model training

We train the weight matrix 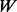 by adjusting 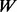 so that 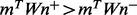, representing the idea that correct name 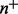 should be ranked higher for the mention 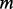 than incorrect name 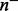. Following (Bai *et al.*, 2010), we use the margin ranking loss (Herbrich *et al.*, 2000), making our model a margin ranking perceptron (Collins and Duffy, 2002). Given 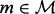, 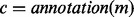, 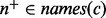 and 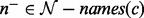, we choose 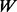 as follows:

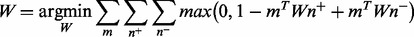



We perform this optimization via stochastic gradient descent (SGD) (Burges *et al.*, 2005). In SGD, a training instance is selected and classified according to the current parameters of the model. If the instance is classified incorrectly, then the parameters are updated by taking a step in the direction of the gradient. In our formulation of pLTR, each instance is a tuple 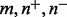, where 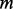 is a mention vector, 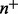 is a name vector that is a correct match for m and 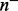 is a name vector that is an incorrect match for m. If 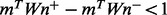, 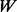 is updated as 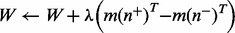, where 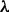 is the learning parameter controlling the size of the change to W. Because SGD is a stochastic method, the order of the training instances is randomized after each iteration; the final 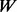 and performance therefore vary slightly.

We evaluated a wide range of values for the learning parameter 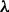 using the development subset of the NCBI disease corpus. We found that while the performance responds to changes in the order of magnitude of λ, it is relatively insensitive to smaller changes (see Section 4 for details). SGD also requires an initial value for the parameters being updated, in this case the matrix 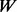. We choose 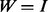, the identity matrix, as the initial value for 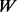, so that the function is initially equivalent to standard cosine similarity.

The number of features in this model is the number of token pairs, 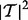. This large capacity makes overfitting a concern. We avoid overfitting through early stopping using the development subset of the NCBI disease corpus as a holdout set. This implies a preference for solutions where 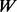 is close to its initial value. We measure performance on the holdout set as the average of the rank of the correct concept for each mention, or 1000, whichever is smaller. We calculate the average rank after each iteration through the training data, and stop training when it increases over the previous iteration.

There are several small differences between our theoretical model and its application. The most significant difference is that our training data are expressed in terms of concepts rather than names. For any given mention 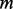 there are typically several names which could be used as 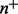, as each concept is usually associated with multiple names. Instead of iterating through all possible combinations of 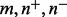, which would be prohibitive, we instead iterate through all combinations of 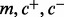, where 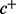 is fixed as 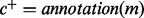 and 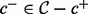. Because we intend the name for 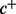 that best matches the mention to be ranked higher than the best-matching name for any other concept 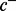, we determine 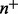 and 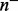 as follows:

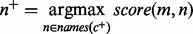


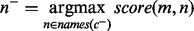



Names associated with multiple concepts do not receive any special handling, however. The second difference is that ∼1.9% of the mentions in the NCBI disease corpus are annotated with a disjunction of multiple concepts. Disjunction annotations, such as ‘D001943|D010051’ for ‘breast or ovarian cancer’, indicate that a single text span contains multiple mentions. We handle these mentions during training by using the original mention as 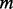 but iterating through the concepts, allowing each to take a turn as 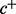. The mention ‘breast or ovarian cancer’ would therefore be used twice, first using 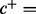 ‘D001943’ and then 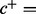 ‘D010051’.

### 2.4 Baseline techniques

We compared DNorm against several strong baseline methods. An exact string-matching method checks for matches of the disease names in text with controlled terminology terms and is therefore expected to have difficulty with term variability, especially if such variations were not foreseen during the creation of the lexicon. In addition, precision may be affected by ambiguous or nested terms. Norm, from the SPECIALIST lexical tools (http://lexsrv3.nlm.nih.gov/LexSysGroup/Projects/lvg/2013/docs/userDoc/tools/norm.html) is a publically available resource of the National Library of Medicine, and is designed to address these issues by normalizing case, plurals, inflections and word order. We used Norm to process all disease names and synonyms in MEDIC and also the set of all strings and substrings of any given PMID document in the NCBI disease corpus. When a text string found in a PubMed abstract in the NCBI testing set was mapped by Norm to a disease name in the MEDIC lexicon, that disease mention is grounded with the corresponding MEDIC concept. For nested disease mentions we kept the longest string that produced a mapping to a MEDIC entry term or synonym. The results of this string matching method are reported as NLM Lexical Normalization in the ‘Results’ section.

Our second baseline method applied MetaMap (Aronson, 2001). MetaMap is another public resource of the National Library of Medicine, and the state-of-the-art natural language processing tool for identifying UMLS Metathesaurus concepts in biomedical text. MetaMap first splits the input text into sentences, and then splits the set of sentences into phrases. For each phrase, MetaMap identifies possible mappings to UMLS based on lexical lookup and on variants by associating a score with each one of them. MetaMap identifies several possible mappings in each phrase and several candidates for each one. In this work, we used MetaMap to identify all UMLS concept identifiers (CUI) in the PubMed abstracts composing the NCBI disease corpus. Then, for each abstract, we used UMLS to map the CUIs to their respective MeSH descriptors and OMIM identifiers. We retained the CUIs we were able to map to either MeSH or OMIM IDs in MEDIC and dropped all others. These results are reported as MetaMap.

We also compare with the benchmark results on the NCBI disease corpus, obtained using the Inference method (Islamaj Doğan and Lu, 2012b). This method was developed on a manually annotated set of PubMed abstract sentences that reflected the consensus annotation agreement of the EBI disease corpus and the AZDC disease corpus (the only available data at the time). The Inference method showed F-measure results of 79%, and it was able to link disease mentions to their corresponding medical vocabulary entry with high precision. Its basis was a Lucene search that first mapped a disease mention against the MEDIC vocabulary. Next, the Inference method makes use of a combination of rules that were used to re-rank the results to report the top ranked one. The core of the Inference method was built as a combination of string matching rules that mapped the text annotated strings to the controlled vocabulary terms. A strong advantage of the Inference method was its incorporation of abbreviation definition detection and the successful use of the fact that the long form of the disease is usually defined elsewhere in the same document. Once the abbreviation was resolved, the knowledge of the mapping of the long form of the disease was used to infer the mapping of the abbreviated mention. To evaluate the Inference method’s performance, BANNER was first applied to each PubMed abstract to identify disease name strings, the Inference method was then applied to normalize each mention to a MEDIC concept.

Our next baseline method uses the same processing pipeline as our DNorm method but replaced our candidate generation method with Lucene, an important component in several previous systems for normalizing biomedical entities (Huang *et al.*, 2011a; Wermter *et al.*, 2009). We loaded MEDIC into a Lucene repository, creating one Lucene document for each concept–name pair. Mentions and names are both processed with the same tokenization and string normalization used in DNorm. A Boolean query is created from the resulting tokens, and the concept for the highest-scoring name is the one returned. We refer to this method as BANNER + Lucene.

Our final baseline method, which we refer to as BANNER + cosine similarity, also uses the same processing pipeline as DNorm. However, this method also uses the same TF-IDF vectors as DNorm for the mentions and names, so that the only difference is the scoring function. The cosine similarity scoring function is as follows:

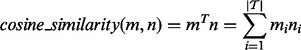

Because this method is equivalent to DNorm with 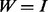, the identity matrix, and 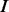 is the value of 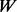 before training, this method isolates the improvement provided by training the 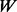 matrix with pLTR.

## 3 RESULTS

During development, all techniques were evaluated using the development subset of the NCBI disease corpus. Varying the learning rate demonstrated λ = 10^−4^ to provide the highest performance on the development set, and this is the setting used for all experiments reported in this section. Final evaluation was performed using the test subset of the NCBI disease corpus.

Our evaluation considers only the set of disease concepts found within each abstract, ignoring the exact location(s) where each concept was found. Thus, the number of true positives in an abstract is the size of the intersection between the set of concepts annotated in the gold standard and the set of concepts returned by the system. The number of false negatives and false positives are defined analogously. Our result measures are precision, recall and F-measure, which were calculated as follows:

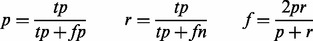



Micro-averaged results were calculated by summing the number of true positives, false positives and false negatives over the entire evaluation set. Macro-averaged results were determined from the number of true positives, false positives and false negatives for each abstract, and the mean result was calculated across all abstracts.

**Fig. 2.**
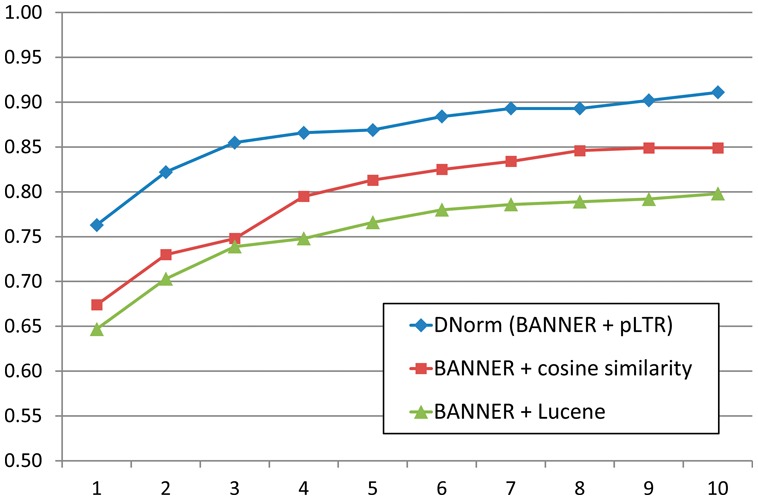
Comparison between BANNER + Lucene, BANNER + cosine similarity and DNorm (BANNER + pLTR) of the micro-averaged recall when considering a concept to be found if it appears in the top n ranked results

Table 2 reports the evaluation results for DNorm and all baseline methods, using micro-averaged performance. Table 3 reports the results for the same experiments using macro-averaged performance. Figure 2 reports the recall for the BANNER + Lucene, BANNER + cosine similarity and DNorm (BANNER + pLTR) experiments if we return more than the highest scoring result from the candidate generation.

**Table 2.** Micro-averaged performance comparing the pLTR method against several baseline approaches, with the highest value in bold

Setup	Precision	Recall	F-measure
NLM Lexical Normalization	0.218	0.685	0.331
MetaMap	0.502	0.665	0.572
Inference method	0.533	0.662	0.591
BANNER + Lucene	0.612	0.647	0.629
BANNER + cosine similarity	0.649	0.674	0.661
DNorm (BANNER + pLTR)	**0.803**	**0.763**	**0.782**

**Table 3.** Macro-averaged performance comparing the pLTR method against several baseline approaches, with the highest value in bold

Setup	Precision	Recall	F-measure
NLM Lexical Normalization	0.213	0.718	0.316
MetaMap	0.510	0.702	0.559
Inference method	0.597	0.731	0.637
BANNER + Lucene	0.662	0.714	0.673
BANNER + cosine similarity	0.692	0.732	0.711
DNorm (BANNER + pLTR)	**0.828**	**0.819**	**0.809**

We created our own implementation of pLTR using the COLT matrix library (http://acs.lbl.gov/software/colt). The implementation enables high performance by taking advantage of the sparsity of the mention and name vectors, training on the NCBI disease corpus training subset in <1 h using a single 2.80 GHz Intel Xeon processor, limited to 10 GB memory. Our implementation scores one mention against the nearly 80 000 names in the lexicon in ∼25 ms using the same equipment. We have applied DNorm to all PubMed abstracts and made the results publicly available in PubTator (Wei *et al.*, 2012, 2013).

## 4 DISCUSSION

Though the NLM Lexical Normalization method has higher recall than any method besides DNorm, the precision remains low because of false positives from phrases such as ‘tumor suppressor’. False positives in the MetaMap results had similar causes; false negatives were frequently due to term variations not present in UMLS or problems with hypernyms, such as mapping ‘autosomal recessive disease’ to Disease (MESH:D004194) instead of the more specific Inborn Genetic Disease (MESH:D030342).

The remaining methods use separate stages for NER and normalization; because all use BANNER for NER, the errors caused by the NER component are the same. The remaining methods also use abbreviation resolution, significantly reducing the number of false positives caused by ambiguous abbreviations. The Inference method handles term variations by using string similarity and Lucene search, though it tends to select highly specific concepts, such as mapping ‘inherited disorders’ to Blood Coagulation Disorders, Inherited (MESH:D025861). Analyzing the errors made by BANNER + Lucene but not by BANNER + cosine similarity shows that most are due to the Lucene scoring function insufficiently penalizing lexicon names containing tokens not present in the mention. The majority of the errors made by BANNER + cosine similarity but not by DNorm are due to term variation.

Because BANNER + Lucene, BANNER + cosine similarity and DNorm (BANNER + pLTR) use the same processing pipeline, the performance difference between these methods is solely due to the normalization methodology. In addition, because the scoring function for cosine similarity is equivalent to the one used by DNorm before training, the performance difference between these methods is solely due to the weights learned during training.

To further isolate the effect of pLTR training on performance, we performed a normalization experiment comparing Lucene, cosine similarity and pLTR using the gold-standard mentions from the NCBI disease corpus test subset as input instead of the mentions found by BANNER. We again used the pLTR model trained using λ = 10^−4^. In this comparison, we count a result as correct if the concept associated with the lexicon name scored highest by DNorm matched the annotated concept for the mention. Out of the 960 mentions, Lucene found 674 (70.2%), cosine similarity found 687 (71.6%) and pLTR found 789 (82.2%). This experiment confirms the effectiveness of the novel learning procedure used by DNorm.

We performed an experiment to demonstrate the effect that varying the learning rate (λ) has on training time and performance. We varied λ exponentially between 10^−2^ and 10^−^^8^, and report the results in Table 4. The best performance was achieved with λ = 10^−^^4^, which required a training time of 48.8 min and resulted in a micro-averaged F-measure of 0.782. While the final performance is similar over a wide range of values for λ, the training time varied widely, ranging from <11 min to >77 h, with smaller values requiring longer training times.

**Table 4.** Effect of varying the learning rate (λ) on the number of training iterations performed, total training time and the resulting micro-averaged F-measure. The highest performance is shown in bold

λ	Iterations	Time (min)	F-measure
10^−2^	4	10.7	0.743
10^−3^	4	13.3	0.765
10^−4^	4	48.8	**0.782**
10^−5^	2	124.0	0.762
10^−6^	8	986.6	0.775
10^−7^	17	4656.5	0.770

### 4.1 Error analysis of DNorm results

**Fig. 3.**
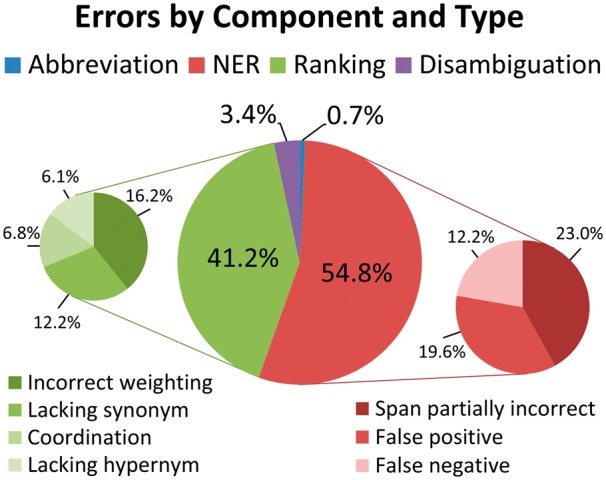
Summary of error analysis. Errors in the NER and ranking components contributed >95% of the total errors

We analyzed the errors made by DNorm, using the model with λ = 10^−^^4^, on the test subset. We considered an error to be either a false positive or false negative; errors were grouped first by the component most responsible for the error and second by the type of error. A chart summarizing the error analysis is presented in Figure 3.

The majority of the errors (54.8%) were traced to the NER component, underscoring the importance of this task in biomedical information extraction. Twenty-three percent of the total errors were due to NER false negatives, predominantly specific diseases (e.g. ‘neisserial infection’) and disease classes (e.g. ‘complement deficiency’), whereas 12.2% of the total errors were due to NER false positives, including ‘molecular defects’, ‘deficiency 879delG’ and ‘cardiac troponin T’, a type of RNA. The remainder of the NER errors, 19.6% of the total errors, resulted from tagging partially correct spans. Examples of the span missing tokens include ‘congenital absence’ instead of ‘congenital absence of the iris’ and ‘breast cancer’ instead of ‘male and female breast cancer’. Errors due to the span capturing extra tokens were less common, as it is easier for the normalization component to recover from extra tokens than missing ones. Examples include ‘paternal uniparental disomy’ instead of ‘uniparental disomy’ and ‘sporadic T-cell leukaemia’ instead of ‘T-cell leukaemia’.

The next largest source of error was the candidate generation using our ranking technique, which contributed 41.2% of the total errors. Of the total errors, 12.2% were due to token pairs not being recognized as having closely related meanings. Many of these were adjective forms, such as ‘cardiac’ meaning ‘heart’ or ‘colorectal’ meaning both ‘colon’ and ‘rectum’. We also found some spelling differences (‘tumour’ versus ‘tumor’) and stemming errors (‘adrenocorticotropic’ stems to ‘adrenocorticotrop-’ but ‘adrenocorticotrophin’ stems to ‘adrenocorticotrophin’).

Unrecognized hypernyms also contributed to the ranking errors, accounting for 6.1% of the total. This is expected because the annotation guidelines for the NCBI disease corpus instructed the human annotators to annotate any mention that does not exactly match a concept in MEDIC with the closest concept that includes it. While some of the unrecognized hypernyms were semantically close, such as ‘hypomania’ being a type of ‘mood disorder’, others were relatively distant, including ‘disorder of glycoprotein metabolism’ being a kind of ‘inborn metabolism error’ and ‘gastrulation defect’ being annotated simply as ‘disease’.

Difficulties in ranking coordinations were the cause of 6.8% of the total errors. These errors are predominantly false negatives because the ranking component only returns the single best disease concept for each mention. For example, in the mention ‘leukemia and/or lymphoma’ the ranking correctly found ‘leukemia’ but missed ‘lymphoma’. In addition, complex coordinations such as ‘breast, brain, prostate and kidney cancer’ occasionally also caused false positives, in this case to ‘prostate cancer/brain cancer susceptibility’ (OMIM:603688).

The greatest number of ranking errors, 16.2% of the total errors, were not attributable to a single qualitative error, but were instead due to an incorrect relative weighting. For example, the mention ‘ptosis’ refers to a drooping of the eyelid, but the method ranked ‘X-linked ptosis’ (OMIM:300245) higher than the correct annotation of ‘eyelid ptosis’ (MESH:D001763). This ranking occurred because ‘X-linked’ is much more common in the lexicon than ‘eyelid’, causing ‘X-linked’ to be given a lower TF-IDF weight, and its absence therefore considered less significant. Another example includes ‘adenomatous polyps’ being matched to ‘adenomatous polyposis coli’ (MESH:D011125) instead of to the disease with the same name (‘adenomatous polyps’, MESH:D018256) because the ranking model learned during training that the token ‘polyps’ is strongly associated with each token in the name ‘adenomatous polyposis coli’.

Only 3.4% of the errors were due to the disambiguation component. An example includes ‘neurohypophyseal diabetes insipidus’, which is a valid name for two concepts that share a parent but are not themselves in a parent–child relationship. Abbreviation processing was the major component that contributed the fewest total errors, 0.7%. An example includes ‘IDMS’, which the abstract defined as ‘isolated DMS’, and where ‘DMS’ had been defined previously as ‘Denys-Drash syndrome’.

### 4.2 Analysis of learned weights matrix

Analyzing the entries in the matrix produced some additional insight into the normalization task and why this technique works. As discussed in the ‘Methods’ section, entry 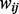 in the weight matrix 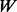 represents the correlation between token 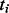 appearing in a mention and token 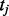 appearing in a concept name from the lexicon. These correlations may be positive or negative. In addition, the matrix is initialized as the identity matrix 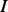, so that non-diagonal entries with a value other than 0 are due to training updates.

The non-diagonal entries with the highest values represent the strongest correlations. As expected, the relationship we found most frequently were synonyms, such as ‘inherited’ → ‘hereditary’ or near-synonyms such as ‘disorder’ → ‘disease’. We also found many entries reflecting other semantic relationships, including hypernymy (‘recessive’ → ‘hereditary’) and others (‘BRCA1’ → ‘ovarian’). We found many examples of terms with morphological variations not handled by stemming, such as ‘gonococcal’ → ‘gonorrhea’ and ‘osteomata’ → ‘osteoma’. We also noted spelling variations, such as ‘haemoglobinuria’ → ‘hemoglobinuria’. Finally, we found many examples of words that appear together frequently (collocations), such as ‘dystrophy’ → ‘muscular’ and ‘disease’ → ‘hereditary’.

We also analyzed the entries in the weight matrix with the lowest values, all negative, representing the strongest negative associations. The most common relationship found always included a head word strongly associated with disease. The head word was typically either paired with another head word (e.g. ‘deficiency’ → ‘infection’) or an adjective (‘abnormal’ → ‘infection’), though others were also observed (‘limb’ → ‘disease’). These relationships suggest the existence of several broad categories of disease, and indicate an attempt to exclude some of these as possibilities. The next most common relationship we found was between words that frequently appear together, or collocations, such as ‘autosomal’ → ‘dominant’. This type of negative correlation reduces the weight of the complete phrase while allowing the weight between each individual token and itself (e.g. ‘autosomal’ → ‘autosomal’) to remain high. We found some evidence of second-order relationships such as ‘fragile’ → ‘linked’, both of which commonly appear with the token ‘X’, as in ‘fragile X’ and ‘X linked’. Thus the pair ‘fragile’ → ‘linked’ reduces the score of a mention containing the phrase ‘fragile X’ with a concept name containing the phrase ‘X linked’. Finally, we also found some antonym relationships, such as ‘dominant’ → ‘recessive’.

### 4.3 Limitations and future work

As the first work to use the pLTR model for normalization, there are remaining questions. While DNorm consists of separate steps for mention and concept finding, this article aggregates the mention-level results into the abstract level for evaluation. Thus additional assessment would be needed when applying DNorm to other text mining tasks such as relationship extraction between gene variants, drugs, disease and adverse reactions (Hakenberg *et al.*, 2012).

While our evaluation only applied DNorm to one dataset, we recently also applied DNorm to the ShARe/CLEF eHealth Task 1b, a disease normalization task in clinical notes involving diseases and disorders from the clinical vocabulary SNOMED-CT (Stearns *et al.*, 2001). DNorm placed first among 17 international teams (Leaman *et al.*, 2013; Suominen *et al.*, 2013). This is encouraging evidence of our method being more generally applicable, though additional evaluation should be performed to verify the effectiveness of our method in other applications, such as full text articles.

Because the scores returned by our model are ordinal values, the model naturally only returns one concept per mention. This poses a difficulty for mentions annotated with more than one concept. We found that the difficulty was not great for mentions annotated with disjunctions, as these often appear independently in other mentions in the abstract. However, an additional 0.5% of the mentions in the NCBI disease corpus are annotated with a conjunction of multiple concepts, indicating that a single mention implies multiple concepts simultaneously. For example, the mention ‘inherited neuromuscular disease’, was annotated as ‘D009468+D030342’ (‘Neuromuscular disease’ and ‘Genetic Diseases, Inborn’). In the present work, conjunction annotations were ignored during training and always counted as false negatives in our evaluation—we made no attempt to give partial credit. While there are relatively few of these mentions in the NCBI disease corpus, additional techniques will be required in tasks where conjunction annotations are critical.

On a more fundamental level, there is no universally agreed definition of disease in general (Scully, 2004). Likewise, specific diseases may be classified differently by different clinicians owing to variations in the presentation of diseases within a syndrome family, differing degrees of granularity or even variations in word meaning (Biesecker, 2005). Even so, disease classifications are constantly being refined, and separating diseases into subtypes can improve the clinical utility of the disease description. There is some evidence of this stratification in the NCBI disease corpus: PMID 9056547 describes a clinically relevant variant of Pelizaeus-Merzbacher disease, for example. While our method does learn the language variations used to refer to diseases, this represents only an early step toward the more difficult problems of handling variations in disease classification or recognizing new subtypes.

Our immediate future work includes applying our method to additional entity types. It would be interesting to compare our technique with existing methods for normalizing gene names. Because the disambiguation step is important for gene names (Lu *et al.*, 2011), we expect it would require a more comprehensive approach than we used here. Because disambiguation is largely orthogonal to the main effort in this article, however, we believe the learning to rank technique may prove useful to gene names as well.

## 5 CONCLUSION

We have shown that pLTR successfully learns a mapping from disease name mentions to disease concept names, resulting in a significant improvement in normalization performance. We have also shown that the training time requirements are modest and that inference time is fast enough for use online. Our approach models many kinds of term variations, learning the patterns directly from training data.

Our error analysis showed that NER is a continued concern, and the analysis of the learned weight matrix showed that morphological analysis is important for this problem. Our technique primarily addresses the candidate generation step in normalization, and could be paired with more sophisticated techniques for disambiguation.

We believe that pLTR may prove to be sufficiently useful and flexible to be applicable to normalization problems in general. While general applicability should be verified in future work, the present article represents an attempt to move toward a unified framework for normalizing biomedical entity mentions with machine learning.
